# Frailty in older maintenance hemodialysis patients: A latent class analysis revealing personalized management

**DOI:** 10.1371/journal.pone.0351155

**Published:** 2026-06-16

**Authors:** Fangping Chen, Xingyue Guo, Dingyuan Wei, Mengxing Wang, Jiayan Wang, Didi Xu, Luyang Jin, Surui Liang, Siyi Li, Xuemei Xian

**Affiliations:** 1 Nursing Department, Sir Run Run Shaw Hospital, Zhejiang University School of Medicine, Hangzhou, Zhejiang, China; 2 Hangzhou Traditional Chinese Medicine Hospital, Hangzhou, Zhejiang, China; 3 School of Nursing, Ma Kam Chan Memorial Building, Hong Kong, China; 4 Shenzhen Hospital of Southern Medical University, Shenzhen, Guangdong, China; Qatar University, QATAR

## Abstract

**Background:**

Elderly patients undergoing maintenance hemodialysis (MHD) exhibit an elevated risk of frailty, which is strongly associated with adverse clinical outcomes, including increased mortality. However, current frailty assessment tools often overlook intrinsic patient heterogeneity, limiting their clinical utility. This study employs latent class analysis to identify distinct frailty profiles in elderly MHD patients, aiming to explore heterogeneity in frailty phenotypes and identify associated factors, thereby informing the development of targeted interventions.

**Methods:**

Data were collected from 227 elderly patients receiving MHD in Guangdong Province, China, from January to April 2023. Participants were assessed using a general information questionnaire and the Fried scale. Latent class analysis was applied to identify subgroups based on frailty characteristics, and Firth logistic regression was used to examine factors associated with frailty class membership.

**Results:**

Two distinct frailty subgroups were identified: Class 1 (low physical activity–high frailty group, 55.1%) and Class 2 (high physical activity–low frailty group, 44.9%). Firth logistic regression demonstrated that combined hypertension (odds ratio [OR] = 0.023; 95% confidence interval [CI]: 0.002–0.119; P < 0.001), older age (OR = 0.746; 95% CI: 0.618–0.840; P < 0.001), longer dialysis duration (OR = 0.935; 95% CI: 0.874–0.976; P = 0.001), lower serum albumin concentration (OR = 0.884; 95% CI: 0.775–0.973; P = 0.010), and lower serum creatinine concentration (OR = 0.996; 95% CI: 0.992–0.999; P = 0.017) were significantly associated with belonging to the high-frailty group. Overweight showed a trend toward a protective association but did not reach statistical significance (OR = 5.874; 95% CI: 0.900–55.438; P = 0.065).

**Conclusions:**

This study highlights the heterogeneity of frailty among elderly maintenance hemodialysis patients. Combined hypertension was strongly and inversely associated with the low-frailty group, indicating its predominance in the high-frailty phenotype. Older age, longer dialysis duration, lower serum albumin levels, and lower serum creatinine levels were significantly associated with the high-frailty phenotype. These findings underscore the importance of personalized frailty assessment and interventions, such as blood pressure management, nutritional support, and tailored exercise programs, to improve health outcomes in this vulnerable population. Future multicenter longitudinal studies are warranted to validate these frailty phenotypes and evaluate their predictive value for clinical outcomes.

## Introduction

Chronic kidney disease (CKD) is an increasing global public health priority linked to significantly elevated morbidity and mortality rates [1,2]. The prevalence of end-stage kidney disease (ESKD), a progressive consequence of CKD, is also increasing [3]. Maintenance hemodialysis (MHD), defined as receiving hemodialysis for more than 3 months [4], serves as a primary renal replacement therapy for ESKD [5], enabling long-term survival.

As the duration of maintenance hemodialysis treatment increases, the incidence of many age-related complications associated with hemodialysis, such as frailty, also increases [6,7]. Frailty is estimated to be present in approximately 14% of the general population [8]; however, it is significantly more prevalent in hemodialysis patients, with a reported range of 33% to 82% [9,10]. Frailty has been identified as a significant risk factor for hospitalization and mortality in patients with ESKD, particularly among the elderly MHD population [6,7]. It has been reported that elderly patients undergoing hemodialysis and who also experience frailty face an approximately 80% increase in mortality risk with advancing age and longer dialysis duration [11]. Frailty is a multifaceted syndrome characterized by a reduction in activity and insufficient response to health stresses, resulting from sarcopenia, susceptibility, and diminished endurance [12]. Various specific factors associated with chronic kidney failure and hemodialysis, such as chronic inflammation, oxidative stress, protein loss, uremic toxin accumulation, and acid‒base imbalance, exacerbate this process [13,14].

The Fried scale, the most widely used scale for measuring frailty, categorizes patients as robust (0), pre-frailty (1–2), or frailty (≥3) based on five criteria: weight loss, slow gait, weak grip, low physical activity, and exhaustion [15]. However, this score-based approach overlooks heterogeneity within frailty phenotypes. For instance, among pre-frailty patients, some exhibit slow gait (indicating musculoskeletal impairment), while others report exhaustion (suggesting metabolic or inflammatory dysfunction) [16]. Such variation hints at distinct underlying pathophysiological profiles, yet remains underexplored.

To address this gap, we applied latent class analysis (LCA), a person-centered method that identifies homogeneous subgroups within populations based on observed characteristics [17]. Unlike variable-centered approaches, LCA captures interindividual heterogeneity and enables the classification of patients into latent subtypes. This study aims to identify distinct frailty profiles among elderly MHD patients using LCA and to examine associated factors, thereby facilitating more targeted and effective interventions for this vulnerable population.

## Materials and methods

### Study design and participants

This cross-sectional study was conducted at Shenzhen Hospital of Southern Medical University, Guangdong Province, China, and adhered to the principles of the Declaration of Helsinki. Ethical approval was obtained from the Ethical Review Board of Shenzhen Hospital of Southern Medical University (Approval number: NYSZYEC20240002). Data collection was performed between January and April 2023. Participants were included if they were: (1) aged ≥60 years, (2) outpatients receiving maintenance hemodialysis (MHD) for ≥3 months, and (3) capable of providing informed consent. The exclusion criteria included (1) a diagnosis of severe psychiatric disorder or (2) significant cognitive or communication impairment. The purpose and procedure of the survey, the contact details of the personnel responsible, and the data security measures were communicated to the participants. Participants who consented to participate signed a voluntary and informed consent document. The confidentiality of the participants was guaranteed by the anonymization of all the data collected. In addition, participants were permitted to disengage from the study at any point without any repercussions.

### Data collection tool

#### Frailty phenotype scale (Fried scale).

The Fried scale, a widely used measure of frailty, assesses five domains (weight loss, slow walking speed, low grip strength, low physical activity, and fatigue), scoring 1 point for each domain met, with a total score of 0–5 (0 = robust, < 3 = pre-frailty, and ≥3 = frailty) [15].

#### Activities of Daily Living (ADL).

The Barthel Index (BADL), a standard tool for evaluating basic ADL, scores activities such as feeding, transferring, dressing, and toileting on a scale from 0 to 100, with higher scores indicating greater independence (20 or lower = total dependence, 21–60 = severe dependence, 61–90 = moderate dependence, 91–99 = mild dependence, 100 = independence) [18,19].

### Data analysis

Using Mplus 8.3, we conducted LCA on the five frailty dimensions. The optimal model fit was determined by the Akaike information criterion (AIC), Bayesian information criterion (BIC), adjusted BIC (aBIC), entropy, Lo–Mendell–Rubin likelihood ratio test (LMR), and bootstrapped likelihood ratio test (BLRT), with lower AIC/BIC/aBIC values indicating better fit and an entropy value close to 1.0 reflecting high classification accuracy. A significant p value (≤ 0.05) from the LMR and BLRT tests indicated that the k-class model provided a significantly better fit than the k − 1 class model. Following LCA, variables associated with class membership in univariate analyses (p < 0.05), together with clinically relevant nutritional and inflammatory markers (albumin, C-reactive protein [CRP], and body mass index [BMI]) [13,14], were entered into the multivariable model. Firth logistic regression was employed to reduce small sample bias and provide stable parameter estimates [20]. Multicollinearity among independent variables was evaluated using variance inflation factors (VIF), with values greater than 10 considered to indicate potentially problematic multicollinearity [21]. As a sensitivity analysis, the results of the Firth logistic regression were compared with those from a conventional binary logistic regression to assess consistency across estimation methods.

## Results

### Characteristics of participants

An effective response rate of 94.58% was achieved by distributing a total of 240 questionnaires, of which 227 were deemed valid. Among the 227 elderly patients who received MHD, the average age was 71.91 years (SD  =  8.08), with males accounting for the majority (59.0%). Among the patients, 136 (59.9%) had low grip strength, and 90 (39.6%) were frail. The detailed categorical characteristics of the 227 participants are listed in Table 1.

**Table 1:** Categorical characteristics of the 227 participants.

Variable	N(%)/M ± SD
**Sex, Male**	134(59.0)^a^
**Age, years**	71.91(8.08)^b^
**Marital status**	
Married	193(85.0)^a^
Widowed	14(6.2)^a^
Single/Divorced	20(8.8)^a^
**Education**	
Primary or below	98(43.2)^a^
Junior high school	79(34.8)^a^
High school	35(15.4)^a^
College or above	15(6.6)^a^
**Medical insurance**	184(81.1)^a^
**BMI**	
Underweight (< 18.5)	138(60.8)^a^
Healthy Weight (18.5 ~ 24)	21(9.3)^a^
Overweight (> 24)	68(30.0)^a^
**Smoking**	
Never	101(44.5)^a^
Quit	79(34.8)^a^
Smoking	47(20.7)^a^
**Drinking**	
Never	103(45.4)^a^
Occasional	95(41.9)^a^
Frequent	29(12.8)^a^
**Dialysis duration, months**	37.68(19.06)^b^
**Dialysis frequency**	
Qw	76(33.5)^a^
Biw	71(31.3)^a^
Tiw	80(35.2)^a^
**Regular dialysis**	211(93.0)^a^
**Dialysis hypotension**	11(4.8)^a^
**Primary disease**	
Diabetic nephropathy	116(51.1)^a^
Secondary glomerulonephritis	33(14.5)^a^
Chronic nephritis syndrome	57(25.1)^a^
Other	21(9.3)^a^
**Combined hypertension**	128(56.4)^a^
**Combined heart disease**	123(54.2)^a^
**Combined diabetes**	106(46.7)^a^
**Polypharmacy, ≥ 5**	128(56.4)^a^
**Frailty conditions**	
Robust	46(20.3)^a^
Pre-frail	91(40.1)^a^
Frail	90(39.6)^a^
**Frailty phenotypes**	
Weight loss	13 (5.7)^a^
Fatigue	127(55.9)^a^
Low physical activity	102(44.9)^a^
Slowed walking speed	93 (41.0)^a^
Low grip strength	136(59.9)^a^
**Barthel index**	
Independence	13(5.7)^a^
Mild dependence	117(51.5^a^
Moderate dependence	38(16.7)^a^
Severe dependence	59(26.0)^a^
**Albumin**	33.87(7.33)^b^
**BUN**	26.36(9.43)^b^
**Scr**	846.67(224.85)^b^
**Ca**	2.30(1.64)^b^
**P**	1.97(0.63)^b^
**K**	5.87(0.79)^b^
**WBC**	7.29(1.90)^b^
**Hemoglobin**	111.16(13.80)^b^
**CRP**	16.73(11.63)^b^

Note. ^a^N (%), ^b^Mean±SD, BMI: body mass index, Qw: once a week, Biw: twice a week, Tiw: three times a week, BUN: blood urea nitrogen, Scr: serum creatinine, Ca: calcium, P: phosphorus, K: potassium, WBC: white blood cell, CRP: C-reactive protein

### Latent class analysis of elderly patients with MHD

#### Results of latent class analysis (LCA).

**Fig 1:**
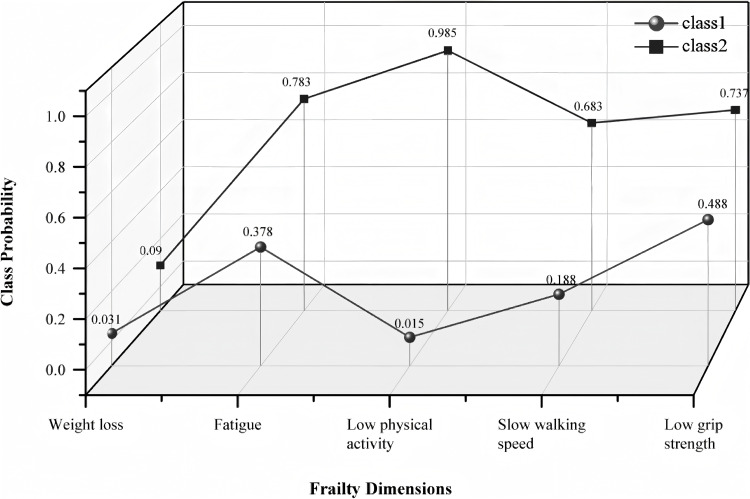
Latent class probability distribution of frailty dimensions in elderly MHD patients. Note. The five dimensions are derived from the Fried frailty phenotype. Class 1 (low physical activity–high frailty), Class 2 (high physical activity–low frailty).

This study employed a total of 1–4 latent profile models, as illustrated in Table 2. The values of AIC, BIC, and aBIC progressively decreased as the number of categories increased from 1 to 2, suggesting a gradual improvement in model fit. When the number of categories was 2, the BIC reached a minimum value of 1247.779, and entropy reached a maximum value of 0.909, while the BLRT (P < 0.001) and LMR (P = 0.0178) were statistically significant, indicating that the 2-category model was significantly better than the 1-category model. When the number of potential categories continued to increase, the BIC value began to increase, indicating that the model fit began to deteriorate, and Entropy = 0.753 < 0.8 when the number of potential categories was 3, indicating a decrease in classification accuracy. Therefore, the 2-category model was chosen as the best potential category model for debilitation in elderly patients with MHD. A potential category was generated based on the classification results, as shown in Fig 1. Compared with Class 2, Class 1 had a significantly greater probability of experiencing low physical activity; therefore, Class 1 was named the low physical activity-high level debilitated group, and Class 2 was named the high physical activity-low level debilitated group.

**Table 2:** Latent class analysis models and fit indices.

Model	LL	AIC	BIC	aBIC	Entropy	BLRT	LMR	Proportions(%)
1C	−668.188	1346.377	1363.501	1347.655	—	—	—	—
**2C**	**−612.889**	**1247.779**	**1285.453**	**1250.591**	**0.909**	**<0.001**	**0.0178**	**55.07/44.93**
3C	−599.420	1232.841	1291.065	1237.187	0.753	<0.001	0.0003	39.21/20.26/40.53
4C	−589.117	1224.233	1303.007	1230.114	0.843	<0.001	0.00138	16.30/38.77/24.67/20.26

Note. Bold values indicate the optimal model.

Abbreviations: K: Number of Free Parameters, AIC: Akaike Information Criterion, BIC: Bayesian Information Criterion, aBIC: Adjusted BIC, LMR: Lo‐Mendell‐Rubin Test, BLRT: Bootstrap Likelihood Ratio Test, —: Not applicable

#### Characteristics of latent class membership.

Age, education, medical insurance status, drinking status, dialysis duration, regular dialysis status, primary disease combined with hypertension, polypharmacy status, blood urea nitrogen (BUN) level, serum creatinine (Scr) level and white blood cell (WBC) count were significantly different between the two latent categories (both p  <  0.05), as demonstrated by the univariate analysis. A greater proportion of patients (55.1%) were debilitated because of low physical activity, with a high level of debilitation. This group was primarily composed of patients who were older, received a lower level of education, had medical insurance, never drank, had a longer dialysis duration, received irregular dialysis, were less likely to have secondary glomerulonephritis, had combined hypertension and were more likely to use more than five medicines at the same time. The high physical activity-low level debilitated group had the highest score in each dimension. The patients in this group were primarily younger, had a higher level of education, had no medical insurance, occasionally drank, had a shorter dialysis duration, were receiving regular dialysis, were more likely to have secondary glomerulonephritis and uncomplicated hypertension, and were less likely to use more than five medicines at the same time. Additionally, the Scr and BUN values of patients in the low physical activity-high level debilitated group were substantially higher than those of the other group. Detailed information is available in Table 3.

**Table 3:** Results of univariate analysis.

Variable	Number	C1(n = 125)	C2(n = 102)	*z/x²*	*P*
**Sex**				*x*² = 1.999	0.157
Male	134	79(63.2)	55(53.9）		
Female	93	46(36.8)	47(46.1)		
**Age, years, M ± SD**	227	76.14(7.25)	66.73(5.68)	*z* = −8.712	<0.01
**Marital status**				*x*² = 4.832	0.089
Married	193	108(86.4)	85(83.3)		
Widowed	14	4(3.2)	10(9.8)		
Single/Divorced	20	13(10.4)	7(6.8)		
**Education**				*z* = 8.632	0.035
Primary or below	98	54(43.2)	44(43.1)		
Junior high school	79	46(36.8)	33(32.3)		
High school	35	13(10.4)	22(21.6)		
College or above	15	12(9.6)	3(2.9)		
**Medical insurance**				*x*² = 5.172	0.023
Yes	184	108(86.4)	76(74.5)		
No	43	17(13.6)	26(25.5)		
**BMI**				*z* = −0.124	0.901
Underweight (< 18.5)	138	75(60.0)	63(61.2)		
Healthy Weight (18.5 ~ 24)	21	13(10.4)	8(7.8)		
Overweight (> 24)	68	37(29.6)	31(30.4)		
**Smoking**				*x*² = 1.637	0.441
Never	101	58(46.4)	43(42.2)		
Quit	79	45(36.0)	34(33.3)		
Smoking	47	22(17.6)	25(24.5)		
**Drinking**				*z = −*2.402	0.016
Never	103	67(53.6)	36(35.3)		
Occasional	95	43(34.4)	52(51.0)		
Frequent	29	15(12.0)	14(13.7)		
**Dialysis duration, months**	227	46.76(17.56)	26.55(14.40)	*z* = −7.972	<0.01
**Dialysis frequency**				*z* = −0.447	0.655
Qw	76	40(32.0)	36(35.3)		
Biw	71	40(32.0)	31(30.4)		
Tiw	80	45(36.0)	35(34.3)		
**Regular dialysis**				*x*² = 4.77	0.029
Yes	211	112(89.6)	99(97.1)		
No	16	13(10.4)	3(2.9)		
**Dialysis hypotension**				*x*² = 0.343	0.558
Yes	11	7(5.6)	4(3.9)		
No	216	118(94.4)	98(96.1)		
**Primary disease**				*x*² = 8.637	0.035
Diabetic nephropathy	116	64(51.2)	52(51.0)		
Secondary glomerulonephritis	33	12(11.4)	21(20.6)		
Chronic nephritis syndrome	57	33(26.4)	24(23.5)		
Other	21	16(12.8)	5(4.9)		
**Combined hypertension**				*x*² = 134.654	<0.01
Yes	128	111(88.8)	17(16.7)		
No	99	14(11.2)	85(83.3)		
**Combined heart disease**				*x*² = 0.215	0.643
Yes	123	66(52.8)	57(55.9)		
No	104	59(47.2)	45(44.1)		
**Combined diabetes mellitus**				*x*² = 0.010	0.921
Yes	106	58(46.4)	48(47.1)		
No	121	67(53.6)	54(52.9)		
**Polypharmacy**				*x*² = 15.884	<0.01
Yes (≥5)	128	84(67.2)	44(43.1)		
No (<5)	99	41(32.8)	58(56.9)		
**Barthel index**				*z* = −1.311	0.190
Independence	13	6(4.8)	7(6.9)		
Mild dependence	117	63(50.4)	54(52.9)		
Moderate dependence	38	17(13.6)	21(20.6)		
Severe dependence	59	39(31.2)	20(19.6)		
**Albumin**	227	34.70(8.05)	32.85(7.24)	*z* = −1.688	0.092
**BUN**	227	28.02(8.754)	24.32(9.868)	*z* = −2.733	0.006
**Scr**	227	982.21(203.40)	680.57(181.00)	*z* = −9.243	<0.01
**Ca**	227	2.19(0.23)	2.45(2.43)	*z* = −0.750	0.453
**P**	227	1.96(0.611)	1.98(0.66)	*z* = −0.270	0.787
**K**	227	5.90(0.80)	5.84(0.78)	*z* = −0.598	0.550
**WBC**	227	7.52(1.81)	7.01(1.98)	*z* = −2.159	0.031
**Hemoglobin**	227	110.46(13.80)	112.02(13.82)	*z* = −0.790	0.430
**CRP**	227	16.99(11.926)	16.40(11.307)	*z* = −0.375	0.708

Note. BMI: body mass index, Qw: once a week, Biw: twice a week, Tiw: three times a week, BUN: blood urea nitrogen, Scr: serum creatinine, Ca: calcium, P: phosphorus, K: potassium, WBC: white blood cell, CRP: C-reactive protein

#### Predictors of latent class membership.

Before the Firth logistic regression, multicollinearity among the independent variables was assessed using VIF. No VIF value exceeded the conventional threshold of 10 (range, 1.06–2.40), confirming the absence of serious multicollinearity among the predictors (Table 1 in S1 File).

The results of logistic regression are presented in Table 4. Combined hypertension emerged as a strong inverse predictor of membership in the low-frailty group (Class 2) (odds ratio [OR] = 0.023; 95% confidence interval [CI]: 0.002–0.119; p < 0.001), confirming its predominance in the high-frailty group. Older age (OR = 0.746; 95% CI: 0.618–0.840; p < 0.001), longer dialysis duration (OR = 0.935; 95% CI: 0.874–0.976; p = 0.001), lower serum albumin level (OR = 0.884; 95% CI: 0.775–0.973; p = 0.010), and lower Scr level (OR = 0.996; 95% CI: 0.992–0.999; p = 0.017) were significantly associated with belonging to the high-frailty group. Overweight showed a trend toward a protective association with the low‑frailty group, although this did not reach conventional statistical significance (OR = 5.874; 95% CI: 0.900–55.438; p = 0.065). Sensitivity analyses comparing the Firth model with a conventional binary logistic regression confirmed consistent directions of association and statistical significance across all variables, indicating that the findings are robust to the choice of estimation method (Table 2 in S1 File).

**Table 4:** Results of Firth logistic regression analysis.

Variable (Class 2 referred to class 1)	*B*	*SE*	*Waldx²*	*P*	*OR*	95% CI
Education (ref:Primary or below)						
Junior high school	−0.253	0.675	0.140	0.762	0.777	0.129 ~ 4.328
High school	0.728	1.031	0.498	0.567	2.070	0.142 ~ 25.603
College or above	−6.720	2.743	6.005	0.129	0.001	0 ~ 1875.156
Medical insurance(ref:No)						
Yes	−1.063	1.067	0.993	0.509	0.345	0.020 ~ 10.733
BMI(ref: Healthy weight)						
Underweight (<18.5)	−0.439	0.919	0.228	0.695	0.645	0.051 ~ 5.467
Overweight (> 24)	1.771	0.767	5.322	0.065	5.874	0.900 ~ 55.438
Drinking(ref:Never)						
Occasional	0.975	0.612	2.538	0.192	2.650	0.604 ~ 13.122
Frequent	1.016	1.057	0.923	0.478	2.761	0.177 ~ 41.694
Regular dialysis(ref:No)						
Yes	−4.865	2.065	5.549	0.103	0.008	0 ~ 511.569
Primary disease(ref:Diabetic nephropathy)						
Secondary glomerulonephritis	0.141	1.045	0.018	0.919	1.151	0.058 ~ 18.950
Chronic nephritis syndrome	0.376	0.870	0.187	0.729	1.456	0.165 ~ 12.666
Other	0.209	0.993	0.044	0.865	1.233	0.111 ~ 18.695
Combined hypertension(ref:No)						
Yes	−3.785	0.785	23.251	<0.001	0.023	0.002 ~ 0.119
Polypharmacy(ref:No)						
Yes	0.994	0.655	2.302	0.207	2.702	0.577 ~ 16.940
Age	−0.293	0.058	26.021	<0.001	0.746	0.618 ~ 0.840
Dialysis duration	−0.067	0.020	11.922	0.001	0.935	0.874 ~ 0.976
Albumin	−0.124	0.044	8.073	0.010	0.884	0.775 ~ 0.973
Scr	−0.004	0.002	8.236	0.017	0.996	0.992 ~ 0.999
BUN	−0.050	0.034	2.084	0.238	0.952	0.869 ~ 1.033
WBC	0.046	0.188	0.060	0.846	1.047	0.648 ~ 1.731
CRP	−0.012	0.025	0.226	0.719	0.988	0.919 ~ 1.054

Note. Firth logistic regression was used to address quasi‑complete separation and small‑sample bias. Wide confidence intervals for certain variables reflect sparse or polarized distributions between frailty classes rather than model instability. BMI: body mass index, Scr: serum creatinine, BUN: blood urea nitrogen, WBC: white blood cell, CRP: C‑reactive protein.

## Discussion

In this study, two frailty phenotypes among elderly MHD patients were identified using LCA. Class 1 was characterized by low physical activity and high frailty. Class 2 was characterized by high physical activity and low frailty. Firth logistic regression revealed that combined hypertension, older age, longer dialysis duration, lower serum albumin concentration, and lower serum creatinine concentration were independently associated with the high-frailty phenotype.

Class 1 accounted for 55.1% of the sample, and Class 2 accounted for 44.9%. Physical activity level was the most discriminating indicator between the two groups. Compared with Class 2, Class 1 showed a markedly greater probability of low physical activity, along with greater impairment related to exhaustion, slow gait speed, and low grip strength. Patients in Class 1 were older, had longer dialysis vintage, had a greater comorbidity burden, and had poorer nutritional status. These patterns highlight the heavy burden of physical inactivity in the MHD population and are consistent with previous reports [22]. However, the frailty prevalence observed in our study was lower than that reported by Ye et al. [23]. This difference may reflect sample characteristics. The participants in that study had longer dialysis exposure and markedly lower physical activity levels. Disease progression and extended dialysis exposure may perpetuate a cycle of physical functional decline through mechanisms such as sarcopenia, chronic inflammation, and metabolic dysregulation [24,25]. Mitochondrial dysfunction and proteolytic activation further contribute to muscle wasting. Individualized interventions are therefore essential [25]. Class 1 patients may benefit from structured exercise programs and nutritional support. Class 2 patients require regular preventive monitoring [26,27]. Patients aged 75 years or older and those with dialysis vintage exceeding 3 years should be prioritized for routine frailty screening.

Combined hypertension was significantly and inversely associated with the low-frailty group, indicating that hypertension was concentrated in the high-frailty group. This finding is consistent with those of previous studies. Xu et al. [28] and Fan et al. [29] reported positive associations between hypertension and frailty risk in elderly inpatients and MHD patients, respectively. Age-related vascular dysfunction, including reduced compliance and autonomic dysregulation, diminishes physiological reserve and accelerates end-organ damage [30]. However, the effect observed here is substantially more pronounced, with a correspondingly wide confidence interval, whereas prior studies generally reported more moderate estimates. This discrepancy likely reflects methodological differences. Previous studies employed conventional regression to directly identify correlates of frailty, where hypertension exhibited a gradient distribution across frailty categories. In contrast, the latent class approach used here first partitioned the sample into two highly differentiated subgroups, resulting in near‑complete separation of hypertension prevalence between classes. The effect of hypertension in the model primarily reflects its ability to distinguish between the two frailty subtypes.

Age and dialysis duration were both independently associated with the high-frailty phenotype. These findings are consistent with those of Bao et al. [31] and Chu et al. [32], indicating that progressive loss of muscle mass and accumulation of chronic inflammation contribute to the development and worsening of frailty. Concurrently, dialysis-induced hemodynamic instability and cardiovascular complications further exacerbate frailty [33]. In the present cohort, the cumulative effects of age and dialysis duration on frailty class membership were substantial, further supporting the use of advanced age and long dialysis vintage as priority triggers for frailty screening.

With regard to nutritional markers, lower serum albumin levels were significantly associated with the high‑frailty phenotype, which is in line with the existing literature [34]. Albumin is a classic marker of protein‑energy wasting in the dialysis population; protein loss during dialysis, chronic inflammation, and metabolic acidosis jointly contribute to hypoalbuminemia, which in turn promotes muscle catabolism and functional decline [34,35]. Lower serum creatinine levels were also associated with the high‑frailty group. As a metabolite of muscle, creatinine is regulated by renal function, skeletal muscle mass, and gut microbial metabolism [36,37]. In dialysis patients, lower creatinine generally reflects reduced muscle mass. No significant association was observed between BUN and frailty class, suggesting that compared with BUN, Scr may be more sensitive to changes in muscle mass in this population.

Overweight showed a trend toward a protective association with the low‑frailty group, although this trend did not reach conventional statistical significance. This trend is consistent with the obesity paradox observed in dialysis patients. A moderately high BMI may provide metabolic reserves and energy storage that buffer against frailty [38,39]. However, BMI cannot distinguish fat from muscle. More precise body composition measurements, such as bioelectrical impedance analysis, are needed to clarify this relationship.

The two frailty phenotypes identified in this study offer a preliminary framework for personalized management. For Class 1, which is characterized by advanced age, long dialysis vintage, heavy hypertension burden, and poor nutritional status, several integrated interventions may be considered. Intradialytic exercise, such as cycling or resistance training, can improve muscle mass and physical function. Regular nutritional assessment and protein-energy supplementation can address hypoalbuminemia and muscle wasting. Individualized blood pressure management with multidisciplinary medication review may reduce the risk of polypharmacy-related adverse events. Nutritional markers and body weight should be monitored monthly. With respect to Class 2, patients currently have relatively preserved functional status. Prevention should be prioritized. Frailty screening with the Fried scale every 3–6 months may help detect early transitions toward the high-frailty phenotype. Moderate physical activity and adequate nutrition should be maintained. The effectiveness of these phenotype-tailored strategies remains to be tested in future randomized controlled trials.

Several limitations should be acknowledged. First, the cross-sectional design precludes causal inference. Second, this was a single-center study in Guangdong Province that used convenience sampling, which may introduce selection bias. Third, some Fried frailty phenotype items rely on self-reports, which carry a risk of information bias from recall difficulties or subjective perception. Fourth, the sample size, although adequate for the LCA, limited the precision of the estimates for covariates with sparse distributions. Fifth, the LCA used only the five physical components of the Fried frailty phenotype. The inclusion of cognitive, psychological, or social dimensions may reveal additional subgroups not captured in this study. Future multicenter longitudinal studies integrating comprehensive geriatric assessments are needed to validate the stability of these latent classes, capture the full spectrum of frailty heterogeneity, and examine their associations with long-term clinical outcomes.

## Conclusions

Using LCA, this cross-sectional study identified two distinct frailty phenotypes among elderly MHD patients: a low physical activity–high frailty group and a high physical activity–low frailty group. Older age, longer dialysis duration, combined hypertension, lower serum albumin concentration, and lower serum creatinine concentration were significantly associated with the high-frailty phenotype. These findings highlight the clinical heterogeneity of frailty and underscore the need for personalized management strategies tailored to different frailty profiles. However, given the single-center cross-sectional design, future multicenter longitudinal studies integrating physical, cognitive, and psychosocial frailty domains are warranted to confirm these phenotypes and evaluate their predictive value for adverse clinical outcomes such as hospitalization and mortality.

## Supporting information

S1 File Multicollinearity diagnosis and sensitivity analysis. (PDF)

S2 File Data. (XLSX)
