# Supplementary material for: Frailty in older maintenance hemodialysis patients: A latent class analysis revealing personalized management
Source: PLoS One. 2026 Jun 16;21(6):e0351155. doi: 10.1371/journal.pone.0351155 (PMC13271483; doi:10.1371/journal.pone.0351155)
Supplement: S1 File — (PDF) [file pone.0351155.s001.pdf]

Table 1 Variance inflation factors for multicollinearity diagnosis

Variable	GVIF	Df	GVIF^(1/(2×Df))
Education	8.188	3	1.420
Medical insurance	1.324	1	1.151
BMI	1.224	2	1.052
Drinking	1.279	2	1.064
Regular dialysis	5.774	1	2.403
Primary disease	2.366	3	1.154
Combined hypertension	1.770	1	1.331
Polypharmacy	1.338	1	1.157
Age	1.521	1	1.233
Dialysis duration	1.452	1	1.205
Albumin	1.188	1	1.090
Scr	1.648	1	1.284
BUN	1.207	1	1.099
WBC	1.197	1	1.094
CRP	1.127	1	1.062

Table 2 Sensitivity analysis: comparison of Binary logistic regression and Firth logistic regression

Variable	Binary logistic regression			Firth logistic regression		
	P	***OR***	**95%CI**	***P***	***OR***	**95%CI**
Class 2 referred to class 1						
Education(ref:Primary or below)						
Junior high school	0.790	0.713	0.059~8.573	0.762	0.777	0.129~4.328
High school	0.559	2.782	0.090~85.860	0.567	2.070	0.142~25.603
College or above	0.986	0.045	0.000~7.209	0.129	0.001	0~1875.156
Medical insurance(ref:no)						
Yes	0.549	4.346	0.035~532.287	0.509	0.345	0.020~10.733
BMI(ref:healthy weight)						
Underweight (<18.5)	0.541	0.353	0.012~9.977	0.695	0.645	0.051~5.467
Overweight (> 24)	0.036	23.537	1.223~453.041	0.065	5.874	0.900~55.438
Drinking(ref:never)						
Occasional	0.130	5.660	0.601~53.275	0.192	2.650	0.604~13.122
Frequent	0.512	3.966	0.065~243.575	0.478	2.761	0.177~41.694
Regular dialysis(ref:no)						
Yes	0.999	0.777	0.000~1.245	0.103	0.008	0~511.569
Primary disease(ref:Diabetic nephropathy)						
Secondary glomerulonephritis	0.995	1.013	0.014~70.964	0.919	1.151	0.058~18.950
Chronic nephritis syndrome	0.999	0.997	0.045~22.310	0.729	1.456	0.165~12.666
Other	0.888	1.312	0.030~57.182	0.865	1.233	0.111~18.695
Combined hypertension(ref:no)						
Yes	<0.001	0.001	0.000~0.035	<0.001	0.023	0.002~0.119
Polypharmacy(ref:no)						
Yes	0.177	5.800	0.453~74.294	0.207	2.702	0.577~16.940
Age	<0.001	0.577	0.436~0.762	<0.001	0.746	0.618~0.840
Dialysis duration	0.004	0.876	0.800~0.959	0.001	0.935	0.874~0.976

Table 2 (Continued)

Variable	Binary logistic regression			Firth logistic regression		
	P	***OR***	**95%CI**	***P***	***OR***	**95%CI**
Albumin	0.014	0.796	0.663~0.956	0.010	0.884	0.775~0.973
Scr	0.013	0.992	0.986~-0.998	0.017	0.996	0.992~0.999
BUN	0.160	0.921	0.820~1.033	0.238	0.952	0.869~1.033
WBC	0.712	1.140	0.569~2.286	0.846	1.047	0.648~1.731
CRP	0.561	0.971	0.879~1.072	0.719	0.988	0.919~1.054
